# Evaluation of carbapenem-resistant Enterobacteriaceae (CRE) guideline implementation in the Veterans Affairs Medical Centers using the consolidated framework for implementation research

**DOI:** 10.1186/s43058-021-00170-5

**Published:** 2021-06-29

**Authors:** Cassie Cunningham Goedken, Marylou Guihan, Charnetta R. Brown, Swetha Ramanathan, Amanda Vivo, Katie J. Suda, Margaret A. Fitzpatrick, Linda Poggensee, Eli N. Perencevich, Michael Rubin, Heather Schacht Reisinger, Martin Evans, Charlesnika T. Evans

**Affiliations:** 1 Center for Access Delivery & Research and Evaluation (CADRE) Center, Iowa City VA Health Care System, 152, 601 Highway 6 West, Iowa City, IA 52246 USA; 2 Center of Innovation for Complex Chronic Healthcare (CINCCH), Edward Hines Jr. VA Hospital, Hines, IL USA; 3 Department of Physical Medicine and Rehabilitation, Northwestern University Feinberg School of Medicine, Chicago, IL USA; 4 Eagle Hill Consulting, Arlington, VA USA; 5 Center for Health Equity Research and Promotion (CHERP), VA Pittsburgh Health Care System, Pittsburgh, PA USA; 6 Department of Medicine, University of Pittsburgh School of Medicine, Pittsburgh, PA USA; 7 Department of Medicine, Division of Infectious Diseases, Loyola University Chicago Stritch School of Medicine, Maywood, IL USA; 8 University of Iowa, Carver College of Medicine, Iowa City, IA USA; 9 Department of Veterans Affairs, VA Salt Lake City Healthcare System, Salt Lake City, UT USA; 10 Department of Medicine, Division of Epidemiology, University of Utah, Salt Lake City, UT USA; 11 Institute for Clinical and Translational Science, Iowa City, IA USA; 12 Department of Veterans Affairs, Lexington VA Medical Center, Lexington, KY USA; 13 VHA MRSA/MDRO Program Office, Lexington VA Medical Center, Lexington, KY USA; 14 University of Kentucky School of Medicine, Lexington, KY USA; 15 Center for Healthcare Studies and Department of Preventive Medicine Institute for Public Health and Medicine, Northwestern University Feinberg School of Medicine, Chicago, IL USA

**Keywords:** Carbapenem-resistant Enterobacteriaceae (CRE), Implementation science, Consolidated Framework for Implementation Research (CFIR), Multi-drug-resistant organisms (MDROs)

## Abstract

**Background:**

Infections caused by carbapenem-resistant Enterobacteriaceae (CRE) and carbapenemase-producing (CP) CRE are difficult to treat, resulting in high mortality in healthcare settings every year. The Veterans Health Administration (VHA) disseminated guidelines in 2015 and an updated directive in 2017 for control of CRE focused on laboratory testing, prevention, and management. The Consolidated Framework for Implementation Research (CFIR) framework was used to analyze qualitative interview data to identify contextual factors and best practices influencing implementation of the 2015 guidelines/2017 directive in VA Medical Centers (VAMCs). The overall goals were to determine CFIR constructs to target to improve CRE guideline/directive implementation and understand how CFIR, as a multi-level conceptual model, can be used to inform guideline implementation.

**Methods:**

Semi-structured interviews were conducted at 29 VAMCs with staff involved in implementing CRE guidelines at their facility. Survey and VHA administrative data were used to identify geographically representative large and small VAMCs with varying levels of CRE incidence. Interviews addressed perceptions of guideline dissemination, laboratory testing, staff attitudes and training, patient education, and technology support. Participant responses were coded using a consensus-based mixed deductive-inductive approach guided by CFIR. A quantitative analysis comparing qualitative CFIR constructs and emergent codes to sites actively screening for CRE (vs. non-screening) and any (vs. no) CRE-positive cultures was conducted using Fisher’s exact test.

**Results:**

Forty-three semi-structured interviews were conducted between October 2017 and August 2018 with laboratory staff (47%), Multi-Drug-Resistant Organism Program Coordinators (MPCs, 35%), infection preventionists (12%), and physicians (6%). Participants requested more standardized tools to promote effective communication (e.g., electronic screening). Participants also indicated that CRE-specific educational materials were needed for staff, patient, and family members. Quantitative analysis identified CRE screening or presence of CRE as being significantly associated with the following qualitative CFIR constructs: leadership engagement, relative priority, available resources, team communication, and access to knowledge and information.

**Conclusions:**

Effective CRE identification, prevention, and treatment require ongoing collaboration between clinical, microbiology, infection prevention, antimicrobial stewardship, and infectious diseases specialists. Our results emphasize the importance of leadership’s role in promoting positive facility culture, including access to resources, improving communication, and facilitating successful implementation of the CRE guidelines.

**Supplementary Information:**

The online version contains supplementary material available at 10.1186/s43058-021-00170-5.

Contributions to the literature

- Implementing guidelines to prevent emerging pathogens builds on strong facility-level culture and infection control practices.
- Our findings suggest access to available resources, effective communication tools, engaged leadership, and strong infection control infrastructure facilitate successful implementation of CRE guidelines.
- Our findings contribute to the implementation literature by emphasizing the importance of local leadership’s role in providing access to available resources as well as promoting guideline implementation.

## Background

*Carbapenem*-*resistant Enterobacteriaceae* (*CRE*) are Gram-negative bacteria (e.g., *Klebsiella* sp. or *Escherichia coli*) that are resistant to most antibiotics. As of 2017, over 13,000 US hospitalized patients had CRE, leading to 1100 deaths and ~ $130 million in healthcare costs [1]. Carbapenemase-producing CRE (CP-CRE) refers to a subset of CRE whose enzymes break down carbapenems, rendering this last line of antibiotics ineffective [2]. CP-CRE infections are currently the main target of prevention efforts as they represent approximately 30% of all CRE, often can share carbapenemase genes with other bacteria leading to spread, and are associated with a greater than 50% mortality rate [3]; thus, CRE/CP-CRE has been designated as an “*Urgent Threat*,” CDC’s highest risk level [1].

The Department of Veterans Affairs (VA) made CRE/CP-CRE prevention a national priority by developing and disseminating guidelines in 2015 to prevent the spread of these organisms [4]. The guidelines were updated in 2017 and 2019 to a directive, to reflect the most current recommendations for CRE/CP-CRE prevention [5, 6]. Key components of VA’s 2015 guidelines/2017 directive include (1) standardizing screening, identification, evaluation, and reporting of CRE, including laboratory testing for CP-CRE; (2) increasing CRE surveillance; and (3) optimizing CRE infection prevention and control in acute care and VA nursing homes (community living centers, CLCs) including use of contact precautions, staff, and patient education materials, and strategies to track cases within and across VA facilities (e.g., interfacility transfer forms) [4–6]. The 2019 directive was published during our evaluation and therefore was not included. VA guidelines and directives provide mandatory national policies and recommendations, but they do not provide additional funding or explicit direction on how best to implement the guidelines at VA Medical Centers (VAMCs). Implementing the new CRE guidelines/directive at VAMCs could be as simple as ensuring that the VAMC had access to PCR testing and that all clinical staff had attended in-service trainings. However, other recommendations might involve purchasing new laboratory testing systems or obtaining access to off-site VA or private sector labs to achieve timely CRE identification to inform clinical decision-making for patients with CRE. To better understand the challenges of consistent widespread guideline implementation across VA facilities, we collaborated with our operations partners in the VHA MDRO Program Office in evaluating VA’s implementation of the CP-CRE guidelines.

MDRO Program Coordinators (MPCs) (similar to an infection preventionist in the private sector) support various Infection Control Program functions (e.g., MDRO surveillance, hand hygiene program, data collection, and reporting) [7] and had primary responsibility for implementing the CRE/CP-CRE national guidelines. Previously published elements of this mixed methods evaluation include (1) an online survey of VA microbiology laboratory staff at 129 VAMCs [8] and (2) an online survey of MPCs at 134 VAMCs with acute care and long-term care units [9].

This paper focuses on the final phase of this mixed methods evaluation by describing results of semi-structured interviews conducted with MPCs and microbiology laboratory staff to identify and understand the contextual factors and characterize best practices and challenges that influenced CRE guideline implementation in VAMCs. We utilized the Consolidated Framework for Implementation Research (CFIR) [10] to analyze the qualitative interview data and examine which CFIR constructs were the most significant contributors to successful implementation of the 2015 guidelines/2017 directive. The overall goals of this qualitative analysis were to determine which CFIR constructs could be targeted to improve CRE guideline/directive implementation and understand how CFIR, as a multi-level conceptual model, can be used to inform guideline implementation.

## Methods

**Fig. 1:**
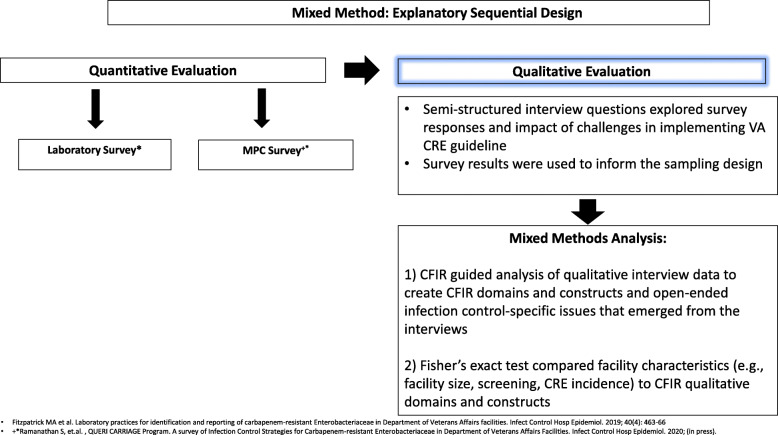
Mixed method: explanatory sequential design

The overall evaluation of the CP/CRE guideline implementation used a mixed methods exploratory sequential design shown in Fig. 1 [11, 12]. In the first phase, an online survey of VA microbiology laboratory staff was conducted and focused on knowledge of CRE guidelines and related laboratory procedures, involvement in guidelines implementation, and comfort executing the guidelines [8]. A parallel survey, addressing knowledge of and comfort with various aspects of the guidelines, was conducted with MPCs at 134 VAMCs with acute care and long-term care VAMCs [9]. The survey results were used to inform the sampling design and interview guides for the qualitative evaluation.

Semi-structured interviews were conducted at 29 VAMCs from October 2017 to August 2018 with MPCs, laboratory staff, infection preventionists, and physicians involved in implementing CRE guidelines at their facility. Interview questions, guided by CFIR constructs, focused on participants’ perceptions of guideline implementation including dissemination, laboratory testing, staff attitudes and training, patient education, best practices, and technology support (e.g., medical record flag, laboratory report/template) (Additional file 1) [10]. Interviewers utilized a templated version of the interview guide for detailed notetaking during all telephone interviews [13]. Notes were also typed directly into the templated interview guide during each interview. The interview team went over the most salient points of the completed interview responses together and saved the collective consensus-based notes. Interviews were also audio-recorded and listened to as needed to ensure accuracy. The consensus-based notes were treated as interview transcripts and uploaded to our secure network for qualitative analysis.

Study sites were selected to include the following factors: (1) geography (rural and urban), (2) VAMC size (e.g., large and small), and (3) VAMC CRE burden (high and low). Characteristics collected about VA facilities were obtained from the VA Office of Productivity, Efficiency, and Staffing (OPES) Facility Complexity Model [14]. VA Corporate Data Warehouse (CDW), a relational database that captures all VA medical encounters, was used to identify the number of CRE-positive clinical or surveillance cultures in 2016. CRE was defined as any culture with *E*. *coli*, *Klebsiella* spp., or *Enterobacter* spp. with non-susceptibility to imipenem, meropenem, or doripenem [5]. Facility-level CRE burden was captured by dividing VAMCs into quartiles using the number of CRE-positive cultures during 2016.

We initially planned to use a stratified random sampling strategy that included self-reported levels of guideline implementation to select sites. However, during the initial interviews, it became evident that while all sites were in the process of implementing the CRE guidelines, participants at sites with no CRE cases could only describe how they might hypothetically deal with CRE. Only sites with any CRE cases had concrete ideas and/or suggestions about improving screening, prevention, or management. CRE screening status of facilities was initially obtained from surveys completed by VA microbiology laboratory staff and MPCs during the first phase of the mixed methods evaluation and clarified in the qualitative interviews [8, 9]. To ensure that we collected the best available information on gaps and/or CRE practices, we oversampled facilities reporting active screening for CRE/CP-CRE and those with more CRE-positive cultures.

Each VAMC has at least one VA funded MPC responsible for implementing the guideline at their VAMC. While all clinical staff at every VAMC have infection control responsibilities, we only interviewed clinicians at each facility (typically 1–2 people) with primary responsibility for ensuring infection control policy development and implementation. In most cases, we interviewed the person(s) who had completed the MPC and/or lab surveys for their VAMC in 2018 or another designee at each site to obtain comprehensive information for each question (see Fig. 1).

Study team members involved in data collection and analysis included experts in implementation science and qualitative researchers (CCG, MLG), social scientists (CB, SR, AV), and infectious disease (ID) experts (epidemiologist (CE), pharmacist (KS), and physician (MF)). Our ID experts participated in the first 6 interviews (14%) to ensure the quality of questioning and prompts. This study was approved by the Edward Hines Jr. VA Hospital Institutional Review Board as a Quality Improvement (QI) study [45 CFR 46.102(d)]. A copy of the Standards for Reporting Qualitative Research (SRQR [15];) checklist is provided in Additional file 2.

### Analysis

We conducted a consensus-based mixed deductive-inductive approach to coding participant responses from semi-structured interviews. Initial analysis of the detailed field notes from the first five interviews was conducted by the analysis team (CB, MG, CCG) and a codebook was developed, using CFIR constructs for guidance [10]. We developed our own codes, primarily to address emergent infection control-specific issues [16]. Each team member initially coded each interview independently and then met as a group to reach consensus. To increase validity and reliability and refining content boundaries of codes and improving coding consistency, 12% of interviews were coded at regular team meetings (MLG, CB, CCG). The remaining interviews (88%) were coded by two team members (CB, CCG), with coding discrepancies resolved with a third team member (MLG). Coding discrepancies involving clinical issues were resolved by ID experts (MF, KS, CE), who also provided clinical interpretations and classified responses as “best practices” if they described unique or innovative strategies. After each response was coded, it was then subsequently rated as positive, negative, or neutral using the criteria shown in Table 1, a modified version of the coding strategy used by Damschroder et al. [17].

**Table 1:** Criteria to rate constructs

+ 1	• Positive influence on the site’s ability to implement the guideline
0	• No influence on the intervention/Describing the sites’ usual practice
− 1	• Negative influence on the site’s ability to implement the guideline

Damschroder et al.’s sample size (*n* = 5) did not allow them to conduct a quantitative analysis of these constructs [17]. Our sample size was sufficient to conduct a quantitative analysis with the CFIR qualitative data to examine factors associated with success in guideline implementation across all CFIR constructs and emergent open codes (codes that did not fall into one of the CFIR constructs). We compared the number of positive vs. negative responses for all CFIR constructs and open codes by whether the sites were actively screening for CRE (vs. non-screening) and by whether the site had any (vs. no) CRE-positive cultures using Fisher’s exact test. Due to the large number of comparisons, we decreased our alpha level to *p* = 0.01 to adjust for multiple comparisons (Table 2).

**Table 2:** Quantitative analysis of CRE guideline implementation by CFIR inner setting constructs and open-code responses^a^

CFIR variables (definitions)	By variable	Total, N (%)	Positive [+], N (%)	Negative [–], N (%)	***P*** value (Fisher’s exact test)	Interpretation
**Comparison of screening vs non-screening sites**						
**1. Leadership engagement** Commitment, involvement and accountability of local leaders and managers with the guideline implementation (*n* = 51)	Not Screening	22 (43.1)	15 (68.2)	7 (31.8)	0.0015	Sites screening for CRE report more leadership involvement in implementing CRE policies compared to sites not screening for CRE, 100% vs. 68.2%
	Screening	29 (56.9)	29 (100)	0 (0)		
**2. Relative priority** CRE is treated as seriously as other health associated infections (*n* = 42)	Not screening	18 (42.9)	10 (55.6)	8 (44.4)	0.01	Sites screening for CRE report CRE is treated as seriously as other HAIs compared to sites not screening for CRE, 91.7% vs. 55.6%
	Screening	24 (57.1)	22 (91.7)	2 (8.3)		
**3. Available resources** Money, equipment, testing supplies, training, education, isolation space, staff time, IT support, and previous workarounds to facilitate guideline implementation are available (*n* = 122)	Not screening	51 (41.8)	23 (45.1)	28 (54.9)	< 0.0001	Sites not screening for CRE report fewer available resources as compared to sites screening for CRE, 81.7% vs. 45.1%
	Screening	71 (58.2)	58 (81.7)	13 (18.3)		
**4. CRE reported incidence episodes**^**b**^ Reported CRE incidence (Y/N) (*n* = 33)	Not screening	19 (57.6)	1 (5.3)	18 (94.7)	0.005	Sites that screen for CRE reported more CRE than sites than non-screening sites, 50% vs. 5.3%
	Screening	14 (42.4)	7 (50)	7 (50)		
**Comparison of sites with CRE cases vs. no CRE cases**						
**1. Communication breakdown**^**b**^ Discussions of team communication or breakdowns (*n* = 53)	CRE	27 (50.9)	27 (100)	0 (0)	0.02	VAMCs with no CRE cases report more communication breakdown than sites with any CRE cases, 100% vs. 80.8%
	No CRE	26 (49.1)	21 (80.8)	5 (19.2)		
**2. Access to knowledge and information** Guideline or training materials (e.g., policies) locally disseminated to relevant stakeholders at each facility (*n* = 25)	CRE	9 (32.1)	8 (88.9)	1 (11.1)	0.016	Sites with any CRE cases report better access to knowledge and information than sites with no CRE cases, 88.9 % vs. 36.8%
	No CRE	19 (67.9)	7 (36.8)	12 (63.2)		

^a^Fisher’s exact test was used to compare the number of positive vs. negative comments for all CFIR constructs and open codes by screening vs. non-screening sites and any (vs. no) CRE-positive cultures

This table focuses on positive results to assist MPCs in implementing the guideline. For example, responses that endorsed a “lack of resources” as a barrier to implementation efforts were coded “positive” (as in “lack of resources was a barrier to implementation of the guidelines”)

^b^Open codes

For this quality improvement funded study, we present positive results to provide guidance to assist MPCs in implementing the guideline. For example, interviewee responses that endorsed a “lack of resources” as a barrier to implementation efforts were coded “positive” (as in “lack of resources was a barrier to implementation of the guidelines”).

## Results

### Participants

We oversampled facilities reporting actively screening for CRE/CP-CRE (9/16) and those with more CRE-positive cultures (quartiles 1 and 2) as shown in Table 3. Forty-three interviews were conducted with microbiology laboratory staff (*N* = 20), MPCs (*N* = 15), infection control nurses (*N* = 5), and physicians (*N* = 3) as shown in Table 4.

**Table 3:** Facility characteristics by CRE incident (quartiles)

CRE ^a^quartiles -->	Q1	Q2	Q3	Q4
CRE-positive cultures (range, N)	16–239 (mean = 46)	7–15	3–6	0–2
Sites reporting screening (*N* = 9)	5	1	3	0
Sites participating in interviews (*N* = 28)	11	7	7	3
Interviewees (*N* = 43)	22	8	7	6

^a^Facility-level CRE burden was calculated by dividing VAMCs into quartiles using the number of CRE-positive cultures during 2016 (*N* = 141)

**Table 4:** Interviewee characteristics

Individuals interviewed	Total participants (***N*** = 43)
Gender	
Male	11
Female	32
Mean years at VA (SD)^a^	12.34 (10.35)
Mean years in current position (SD)^b^	6.26 (6.11)
Occupation	
Microbiology Laboratory Staff	20
Multi-drug-Resistant Organisms Prevention Program Coordinator (MPC)	15
Infection Control Nurse	5
Infection Control Physician	3

^a^Missing 1 observation

^b^Missing 2 observations

### CFIR domains and constructs: categorizing coded responses

**Fig. 2:**
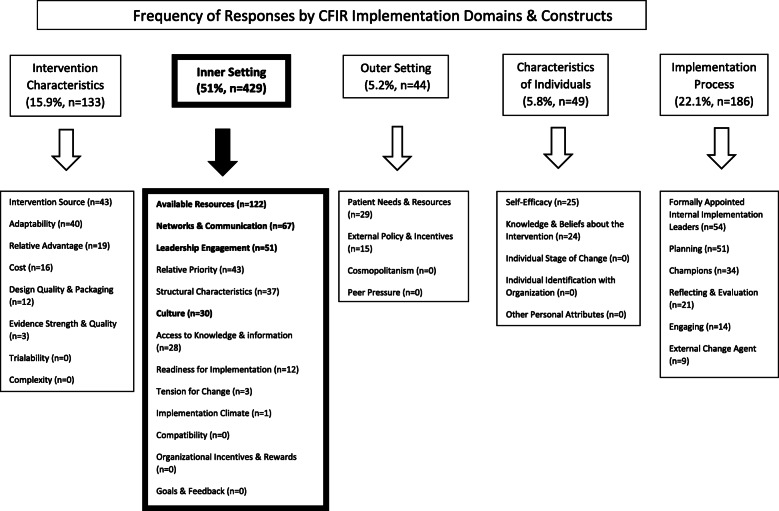
Frequency of responses by CFIR implementation domains and constructs

In Fig. 2, we present the 841 interview coded responses categorized by CFIR domains related to system-wide CRE guideline implementation: (1) *inner setting* (*n* = 429, 51%), (2) *implementation process* (*n* = 186, 22.1%), (3) *intervention characteristics* (*n* = 133, 15.9%), (4) *characteristics of individuals* (*n* = 49, 5.8%), and (5) *outer setting* (*n* = 44, 5.2%) (see Additional file 3 for CFIR domain and construct definitions).

### CFIR domains and constructs: examining associations

In Table 2, we show results of selected quantitative analyses comparing CFIR constructs and open codes by whether the sites were actively screening for CRE (vs. non-screening) and any (vs. no) CRE-positive cultures. Our analysis identified the following constructs as being significantly associated with CRE screening or presence of CRE: *leadership engagement*, *relative priority* (“CRE is as important as other Hospital Acquired Infections (HAIs)”), *available resources* (e.g., IT support, staffing), *team communication* (operationalized as communication breakdown), and *access to knowledge and information* (e.g., laboratory/clinical staff and patient/family educational material).

VAMCs that were actively screening for CRE reported significantly more leadership engagement in implementing CRE policies than those VAMCs that were not screening, 100% vs. 68.2% (*p* = 0.002). Sites screening for CRE reported that it was treated as seriously as other HAIs (e.g., with methicillin-resistant *Staphylococcus aureus* (MRSA)) when compared to sites not screening for CRE, 91.7% vs. 55.6% (*p* = 0.01). Sites not screening for CRE were significantly more likely to report a lack of available resources (compared to sites screening for CRE) 81.7% vs. 45.1% (*p* < 0.0001).

VAMCs with any CRE cases (vs. no CRE cases) were less likely to report team communication breakdowns than sites with no CRE, 100% vs. 80.8% (*p* = 0.02). Sites with any CRE reported better access to knowledge and information compared with sites with no CRE, 88.9% vs. 36.8% (*p* = 0.02).

### Understanding the multi-level aspects of CRE guideline implementation

Results of qualitative analyses include findings from representative responses coded by CFIR domains and constructs (Fig. 2) as well as emergent open-coded responses identified during analysis, including responses categorized as “best practices” (defined as an innovative approach to implementation or guideline recommendations).

Within the *intervention characteristics* domain, responses categorized within the *intervention source* construct described which CRE guidelines participants reported using (e.g., VA, CDC, state), with most reporting using the VA’s CRE guidelines. We coded responses that described adherence to CDC or state (but not VA) guidelines as negative. Best practices examples involved sites adapting or modifying current MDRO policies to include recommendations from the VA CRE guideline.

Within the *outer setting* domain, *patient needs and resources* construct was defined as strategies and/or materials used to educate or engage veterans and their families. Positive responses focused on the adequacy of and access to adequate educational materials to inform patients and their families about CRE (including the availability of materials in various languages and literacy levels). Negative responses described sites lacking knowledge of or access to handouts for educating laboratory and/or clinical staff and patients and families. An MPC described it this way:

It would be nice to have [more educational] materials [and staff release time for CRE-related training] (MPC).

Best practices described strategies to promote staff training, development of lab/clinical staff, and patient and family educational materials, including educational brochures targeting those with low literacy.

Within the *characteristics of individuals* domain, *self*-*efficacy* was defined as the participant’s confidence in understanding and implementing CRE guidelines. Most participants’ self-efficacy was high, reporting feeling “very” or “fully” confident in implementing the guidelines. The negative response described existing challenges in cohorting MRSA patients and being unsure of how to address CRE patients. Best practices included protected time for a dedicated person to monitor CRE at their VAMC.

Within the *implementation process* domain, the *formally appointed internal implementation leaders* construct was defined as participant’s existing infection control structure (units/departments/committee/resources) and/or laboratory testing of other MDROs. Negative responses centered on gaps in existing MDRO reporting strategies (e.g., overreliance on verbal vs. electronic communication). Best practices described a strong infection control structure based on good communication that enables sites to easily notify infection control team members of a CRE-positive patient. Commercially available software (e.g., Theradoc®) was also described as facilitating staff communication.

Table 5 describes CFIR *inner setting* domains, definitions and representative positive and negative as well as best practices responses. Just over half of all coded responses addressed constructs within the *inner setting* domain (e.g., factors promoting successful CRE guideline implementation). Because these factors are potentially modifiable and are important for addressing effective implementation, four of the top six *inner setting* constructs are highlighted (Table 5): (1) *available resources* (*n* = 122, 28.4%), (2) *networks and communication* (*n* = 67, 15.6%), (3) *leadership engagement* (*n* = 51, 11.8%), and (4) *culture* (*n* = 30, 6.9%).

**Table 5:** Representative positive, negative, and best practices responses for CFIR inner setting domains for CRE guideline implementation

CFIR domains	Representative responses		
	Positive	Negative	Best practices
**1. Available resources** **Definition**: Money, equipment, testing supplies, training, education, isolation space, staff time, IT support, and previous workarounds to facilitate guideline implementation are available	*IT support and staffing*: (…) we have a CPRS [discharge] template. [CRE status] would be communicated in the template and the nurses [also] call the accepting facility and give a [verbal] report. [MPC] *Timely lab reporting*: The [CRE testing] cartridges last about 4-6 months, (…) and if I only get 3 [CRE cases] within those 6 months, then I have to throw the other 7 cartridges away. [By comparison, the University lab can provide] results in] …about 24 hours. [Laboratory]	*Lab testing equipment and training*: [New lab equipment has to be purchased and time has to be allocated for staff to be trained and be proficient.] It’s hard to add new testing in the VA. [Laboratory] *Staffing*: [We] had a lot of [staff] turnover in infection control for a long time, and when you have less experienced with] less training, then that’s a challenge. [*Infection Control Staff*] We just don’t have the staff [to implement CRE screening] right now. [MPC] *Educational materials/Staffing*: It would be nice to have [more educational] materials [and staff release time for CRE-related training]. I tried … to do an in-service every 30 minutes and [only] had 5 people that day. [MPC] *Isolation beds*: [Our] spinal cord [unit] is a problem because they [only] have four bed room[s] [individual isolation rooms are not available]. That’s an infection control issue if someone is [CRE] positive. [MPC] *IT support*: The handoff communication is verbal and the transfer note doesn’t [routinely address the patient’s] isolation status. When I call the new unit, [unless] they check their Theradoc ® … they won’t know. [MPC]	*Staff training workaround*: [In addition to] unit-based training, [we] developed poster boards [for] the Infection Control week. After [the] initial training, physicians were still ignoring and bypassing order sets. [So] posters [were hung] in meeting rooms and any rooms where physicians did their documentations. They finally are getting numbers that are representative. [Infectious Disease Chief] *Training and materials*: The acute care people do not think it’s important to have a full class. … I do a train-the-trainer session [now]. [MPC] *Lab reporting*: But since [our lab] system was updated with the new isolates and packages, we are now able to select the CRE organism. We are doing active surveillance and Infection Control developed a list of high-risk patients and recommendations for screening from the guidelines. [Laboratory] If a [CRE+] patient is… flagged from a previous encounter, …they are automatically put in isolation on admission [based on] the Theradoc® documentation. [Laboratory]
**2. Networks and communication** **Definition**: Local infrastructure for national guideline or training material dissemination (e.g., CRE-related conference calls to train staff).	[Our] electronic communication includes… a daily print out… an email group … [for] asking questions and vice versa … [and] hard copies of [overnight] test results. There may [also] be a phone call or a voicemail. It’s [a] multi-prong approach. … We have pamphlets online that they [staff] can print if we [Infection Control] aren’t around. We try to coach them to be self-sufficient. [Infection Control Nurse] [Guideline information is disseminated] through [the] MDRO group, … front office … [and] the VISN. [Staff] attend [the] Antimicrobial Stewardship Program interdisciplinary committee meeting, …staff meetings, … [and receive] email from the IP doctor. [MPC]	There is a lag in the policy being implemented once published. [I] didn’t get [timely MPC-level] access to the [MDR Office] communications, emails, and calls. [Infection Control Manager] There were a lot of delays here, … outdated policies and possibly didn’t rise to the top as priorities], [causing] a lag in communications… [So, guideline] issues haven’t been brought up [to VAMC and VISN] Infection Control yet. [Infection Control Manager]	I also created an [CP-CRE] algorithm … [that] informed everyone …who to test, what to do, and when to contact Infectious Disease. There is also a binder on every unit where everything sits. [MPC] I go to [the units] and make sure they have all signs up, … [and that] they have … [participated in a CRE] in-service. I asked them to educate the patient … or I’ll do it with them if they aren’t comfortable. I also talk to the attending and resident physician as well. Some don’t understand the need for isolation, so it’s important we speak to everyone. [MPC]
**3. Leadership engagement** **Definition**: Commitment, involvement and accountability of local leaders and managers with the guideline implementation.	[If we tell leadership what’s needed], they’ll say okay and get it for us. If we started seeing more CREs, and we need [new testing equipment], they’ll find the money to support it. [Laboratory] Laboratory [leadership handles all] equipment needs … and any new assays. [Infection Control Staff] [Based on previous experience,] we go to [leadership] saying we need … [lab equipment] and they’ll say okay and get it for us. [Laboratory]	[Leadership] allowed us to do a CPRS flag and … to send out confirmation testing and … set up send out protocols. However, it takes a long time to get this much done. [MPC]	[Our] Medical Director is also very involved in Infection Control and Infectious Disease… [and] works with [us] on projects that arise. [MPC] We’ve implemented a great [computer] tool … for screening admissions for [CRE] risk factors and [are] implementing … swabbing for those with risk factors (e.g., international travel, organ transplant recipients). [MPC]
**4. Culture** **Definition**: Infection Control policies already implemented locally or in anticipation of new MDROs.	I think our facility takes a very strong stance on minimizing exposure. In general, the culture here is very aware of infect[ious] agents and how to prevent spread. The facility [has only private rooms, which] helps. [Laboratory] We don’t have a big problem with it [CRE] yet. It’s the new emerging pathogen and we are giving it a lot more press. I think the [staff has] … a good attitude. [MPC]	Some people do or don’t see or follow [existing MDRO practices, like] hand hygiene and PPE appropriately, all the time for a variety of reason[s]. [MPC] [Providers] think it’s ridiculous, that we require contact precautions. [MPC]	[At the monthly Infection Control meeting], we always make sure to talk about [CRE] and what new processes we need to implement or … improve. [Laboratory] Every new [lab] person has to rotate through microbiology. [Laboratory] We’ve done an excellent job with MDROs at the facility, people are very cooperative and enthusiastic. [Our] MRSA [rate] is dropping rapidly. People are really working hard …with Infection Control … to clean rooms, …come to the Infection Control meetings, and there [are] always questions. The [Infectious Disease] Chief does a lunch and learn… every month. He answers any questions about infection control. [Laboratory]

### Inner setting constructs

#### Available resources

Successful implementation of the guidelines begins with stakeholders’ perceptions of the immediacy of the problem. One interviewee described trying to implement the guidelines as very challenging in the absence of any CRE cases:

It’s like waiting for it to snow in Florida. You’re doing preparedness training [but] people don’t listen and … [they don’t] think it’s real. Because they haven’t seen it … People don’t begin to be proficient unless they come in contact with it. [Laboratory Staff]

Participants at sites with CRE cases indicated that successful implementation of the CRE guidelines required access to and/or deployment of various resources (e.g., staffing, acquisition of new laboratory equipment to conduct polymerase chain reaction [PCR] testing, and/or information technology). One MPC described obtaining necessary resources in the following way:

[Our] Chief of Staff… did go to [the] chiefs of various services and said to them, you need to take the CRE screening seriously. Leadership certainly pushed all the physicians to implement the guidelines. If we … had more positive CRE [cases], I’m positive they [leadership] will be on top of it. [MPC]

At sites where the guidelines were not fully implemented, participants described inefficient or overly complicated processes for obtaining new laboratory equipment and/or addressing staff turnover, insufficient contact isolation rooms, and/or dependence on unreliable strategies (e.g., word-of-mouth) for communicating patient CRE status.

Best practices for the *available resources* construct included strategies to enhance dissemination of CRE educational materials to providers (e.g., posters and train-the-trainer in-services), timely access to laboratory reports to inform clinical decision-making, and more formal strategies to systematically communicate patients’ CRE test results (positive or negative) to all relevant parties (e.g., admission/discharge templates in the electronic health record [EHR]).

To confirm CP-CRE, all VAMCs who did not have the available equipment were required to purchase new laboratory equipment or to identify an outside laboratory with PCR testing capacity. Once a potential case of CP-CRE was identified, participants described ongoing monitoring of confirmation test results of CP-CRE and developing strategies to ensure adequate isolation space to keep the identified individual in contact precautions until CP-CRE status was confirmed.

#### Networks and communication

Coded responses described positive and negative aspects regarding CRE guideline dissemination. Sites reporting good communication described adapting a robust pre-existing infection control infrastructure to successfully address CRE. Other sites cited overly complicated and/or hierarchical bureaucracy as hindering dissemination by contributing to delays in development or approval of local policies and impeding CRE guideline implementation.

Best practices for this construct included employing strategies, tools, and innovations to facilitate local guideline implementation. One successful strategy described including CRE in existing MDRO policies versus developing a CRE-specific policy, which often delayed guideline implementation.

Participants most commonly reported learning about the VA CRE Guidelines through one or more national, regional, and local communication mechanisms, including hospital and discipline-specific (e.g., laboratory, infectious disease, pharmacy) email listservs. Guidelines were also discussed and disseminated at local in-service trainings, new employee orientation, and/or ad hoc trainings (e.g., daily patient rounds), as well as during local interdisciplinary committee meetings (e.g., antibiotic stewardship committee meetings).

#### Leadership engagement

*Leadership engagement* was defined in terms of local leaders’ commitment to, involvement in, and accountability for CRE guideline implementation. Sites successfully implementing the CRE guidelines commonly cited local (VAMC, laboratory, and/or infection control [IC]) leadership engagement in facilitating timely procurement of laboratory testing equipment. Negative responses described local (VAMC, laboratory, and/or IC) leadership as “uninvolved” in guideline implementation and/or unwilling to address adequate isolation space, staffing to conduct CRE testing, release time for staff education, or funding to purchase laboratory equipment. Best practices described active leadership engagement in guideline implementation and participation by all relevant clinical and laboratory staff.

#### Culture

*Culture* responses were classified as “positive” when they described strong local infection control practices, a proactive approach to CRE prevention and/or management, and/or heightened awareness of infection control and prevention for emerging pathogens (e.g., active screening for CRE in high-risk patients or a plan to incorporate active screening into current practices if or when CRE incidence increased). “Negative” culture responses described local knowledge about CRE as lacking and/or poor staff compliance with hand hygiene and/or use of personal protective equipment (PPE) (gowns or gloves):

It’s very challenging, I’ve been here a year. When I do environmental rounding and corrections, if someone is not [using] PPE for example, [I get] a lot of push back. I think [staff] think CRE is serious, because I’ll go talk to them face-to-face, if someone has a history of it. But MRSA, isn’t treated as seriously. [MPC]

Best practices examples for culture addressed interdisciplinary collaboration, education, and/or cross-training of laboratory staff on microbiology/ infection control practices.

## Discussion

Healthcare-associated infections are common, with greater than 1 million occurring annually across the US healthcare system, representing an ongoing threat to patient safety [18]. In 2020, in comparison with other MDROs, CRE remains relatively uncommon in VAMCs. A subset of CRE, CP-CRE, is particularly worrisome as they may be responsible for increasing CRE spread in the USA [1] and mortality rates due to CP-CRE remain high [19–21]. Thus, VA is implementing guidelines in all acute care and skilled nursing facilities to prevent further spread of CRE. Nearly all survey respondents reported using VA CRE/CP-CRE guidelines, most with high confidence (79%) [9].

Effective CRE identification, prevention, and treatment require ongoing collaboration between clinical, microbiology, infection prevention, antimicrobial stewardship, and infectious diseases specialists. Evidence suggests that prevention can be strengthened by using standardized tools that facilitate effective communication as well as identify gaps in policies/procedures (e.g., electronic admission and discharge templates, patient flags, protocols, and strong IC organizational culture) [22–26].

CFIR was used to code interview responses in order to better understand how guidelines were implemented and to guide creation of case memos disseminated to MPCs to facilitate use of “best practices” to implement the guideline (see Additional file 4). Based on our analyses, CRE incidence plays an important role in increasing awareness and willingness to take steps to fully implement the guideline. While VA actively screens all new admissions for other MDROs (e.g., MRSA), CRE screening remains uncommon at most VA facilities. In the absence of any CRE incidence at a given facility, guideline implementation was described as very challenging. During our initial interviews, it became clear that the experience of even one CRE case strongly increased staff perceptions of the importance of implementing the guidelines and overall priorities for CRE at their facility. This was confirmed in our quantitative analyses, where having any CRE-positive cultures was associated with helping sites to identify and address potential gaps in existing policies and/or procedures.

CRE prevention and management builds on strong infection control practices and facility culture. Our results indicate that existing MDRO policies and practices may need to be modified to address CRE-specific issues of identification, prevention, and/or management. For example, available resources were identified as crucial for the successful implementation of the CRE guidelines. Even sites that reported success in implementing the guidelines requested more standardized CRE prevention and management educational materials for (1) laboratory staff, (2) clinical staff, and (3) patient and family members. Research supports the idea that for infection prevention practices to be successful, healthcare workers must receive ongoing infection prevention specific education [27–32]. Participants also identified the need for training materials for new and existing clinical staff and dedicated training time to ensure that all clinicians understood relevant risk factors, prevention, and management strategies for patients with CRE. The need for educational materials, especially for patients and families, is well established within the healthcare setting [33]. In particular, requests were made for CRE-specific patient/family education materials for staff to facilitate obtaining consent to collect rectal swabs. VA MDRO Program Office has provided virtual training, staff and patient/family educational resources, and shared documents via SharePoint.

The empirical evidence supporting the efficacy of education, by itself, remains weak. Research suggests that education may be more effective in promoting behavior change when combined with other strategies [34, 35]. We and others found many other factors, including culture and environment, management structures, and resources are also needed for an organization to successfully implement guidelines [36].

We previously found that strong leadership support was essential and specifically identified as a facilitator for successful implementation of the MRSA infection prevention guidelines in spinal cord injury and disorders settings across VAMCs [37]. These findings also highlighted the importance of strong local leadership, particularly VAMC laboratory and/or infection control leadership, on the success of CRE guideline implementation. Leadership is key in providing adequate resources [27] and helping shape positive workplace/facility culture [35, 38, 39]. These current results emphasize the importance of local leadership’s role in promoting positive facility culture which may include healthcare worker’s heightened awareness of infection control and prevention for emerging pathogens, facilitating successful implementation of the CRE guidelines.

At the time the guidelines were disseminated, the MDRO Program Office held multiple online training sessions, including real-time question and answer sessions as part of their regularly scheduled monthly MPC calls. Our interview guide included questions about CRE guideline dissemination, in-person conferences and education/training materials. While we did not specifically ask about perceptions of the ongoing MDRO Program Office training program or access to training materials, some participants, primarily MPCs, described a lack knowledge of the availability of training and educational resources to support local guideline implementation. It is unclear how local leadership support and dedicated resources are related to MPC’s perceived lack of knowledge of available national resources. However, issues of staff turnover, VA’s shift from large in-person conferences to virtual training, context, and culture likely contribute.

Changes in clinical practice rely on both the nature and strength of the evidence as well as the setting in which the practice is to be placed, including the way that the process is facilitated [40, 41]. Further, implementation interventions generally target patient-, provider-, and/or system-level behavior change [34]. Responses classified as contextual factors described a variety of factors associated with successful implementation of the CRE guideline. Some contextual factors are easier to change than others. For example, increasing access to isolation rooms for CRE-positive patients is challenging to address, especially in older hospitals, with multi-bed rooms. Less concrete factors, like culture, can be more open to change when a critical mass of individuals perceive an immediate threat. This threat can then push people to very quickly change what might have previously been seen as unchangeable.

One of the most urgent contextual factors within the CFIR networks and communication construct was the need for proactive, consistent information exchange about patient CRE status across all clinical providers and settings. Effective communication can interrupt CRE spread, highlighting the necessity of information technology to support reporting of patient CRE/CP-CRE status within and between settings. Prompt and accurate identification of and communication about patient CRE status at admission and discharge is vital for improving infection control outcomes [22, 23, 42–44]. Our findings support the potential for standardized laboratory, admission, and discharge templates to improve and communicate patient CRE status.

### Limitations

This study was conducted in VA, and perceptions and practices may be different in the private sector or non-US hospitals. In addition, we used a cross-sectional study design during a dynamic period of change in practices to address CRE. Many of these practices and perceptions may likely have been addressed or changed since our interviews were conducted.

## Conclusions

CRE/CP-CRE incidence is increasing and it has spread throughout the USA, with the potential to spread within all acute and long-term care facilities. Controlling CRE spread and improving patient outcomes requires timely and reliable detection and identification methods, followed by effective antibiotic treatment and implementation of enhanced infection control practices. Additional research is needed to examine how best to develop and implement guidelines to address the “imminent vs. looming threat” of new and rapidly developing MDROs for the fields of infection prevention as well as implementation science. Continued efforts are needed to understand participants’ perceived lack of knowledge of available training and educational resources and work to develop targeted implementation strategies to bridge gaps to strengthen VA CRE/CP-CRE prevention efforts.

Sites successfully implementing CRE guidelines described having access to available resources, effective communication tools, and strategies and engaged leadership, as well as a strong infection control infrastructure. Further, CRE/CP-CRE prevention requires a comprehensive multi-faceted approach to interrupt its spread including access to adequate resources and communication capabilities. CFIR posits two constructs within the inner setting domain (leadership engagement and available resources) operate as independent factors. However, in our study, access to necessary resources occurred almost exclusively through local leadership engagement. In addition to local leadership’s role in providing access to resources, they were also described by sites with any CRE incidence as promoting positive facility culture, increasing healthcare workers’ awareness of infection control, prevention of emerging pathogens, and improving communication and facilitating successful CRE guideline implementation. Our findings raised question about how to better understand the intersection of available resources and leadership engagement to improve guideline implementation efforts. Our contribution to the implementation science literature was in demonstrating a significant association between VAMC structural characteristics (e.g., CRE burden) and CFIR constructs (from the interviews). Future evaluations of guideline implementation are needed to elucidate the relationship between leadership engagement and available resources.

## Supplementary Information

**Additional file 1.** MPC/Infection Control Interview Questions on CRE Guidelines.

**Additional file 2.** Standards for Reporting Qualitative Research (SRQR).

**Additional file 3.** CFIR Constructs and Construct Definitions.

**Additional file 4.** MPC Interview Case Memos: Support and Enhancement of VA Guidelines to Prevent the Spread of Carbapenemresistant Enterobacteriaceae (CRE) Qualitative Interview results.

## Data Availability

The datasets generated and/or analyzed during the current study are not publicly available due to participant privacy but are available from the corresponding author on reasonable request.

## Abbreviations

VHA: Veterans Health Administration
VAMCs: Veterans Affairs Medical Centers
HAIs: Healthcare-acquired infections
VA: Veterans Affairs
MDRO: Multi-drug-resistant organisms
MPCs: Multi-Drug-Resistant Organism Program Coordinators
CRE: Carbapenem-resistant Enterobacteriaceae
CP-CRE: Carbapenemase-producing CRE
