# Supplementary material for: Evaluation of carbapenem-resistant Enterobacteriaceae (CRE) guideline implementation in the Veterans Affairs Medical Centers using the consolidated framework for implementation research
Source: Implement Sci Commun. 2021 Jun 29;2:69. doi: 10.1186/s43058-021-00170-5 (PMC8243642; doi:10.1186/s43058-021-00170-5)
Supplement: Supplementary file 1 — Additional file 1. MPC/Infection Control Interview Questions on CRE Guidelines. [file 43058_2021_170_MOESM1_ESM.pdf]

MPC/Infection Control INTERVIEW QUESTIONS ON CRE GUIDELINES
VA QUERI CARRIAGE Program
Interview about Infection Control Practices Related to Carbapenem-resistant Enterobacteriaceae (CRE)

These interviews are focused on understanding infection control practices related to CRE in your facility. The VHA 2017 Guideline for Control of Carbapenemase Producing-Carbapenem Resistant *Enterobacteriaceae* (CP-CRE) is focused on CP-CRE, but we are also interested in learning about practices related to the identification, surveillance, and control of all CRE. The interviews will help us to understand current implementation of the VHA CP-CRE guidelines and to identify best practices and recommendations to support implementation across VA facilities. You are being asked to participate because of your role in CRE surveillance practices and procedures.

Demographics

1. What is the title of your position?
2. How long have you been working in the VA?
3. How long have you been working in your current position?
4. What percentage of your time would you estimate is spent on direct patient care (vs. administrative, teaching, others)?
5. Gender:

Guideline Related Questions

6. Experience with CRE: Please tell me a little bit about your facility's experience with CRE/CP-CRE.
    a. To what extent is CRE a problem at your facility? (If possible, prompt using the list below)
    b. What about other facilities in your community?
    c. Other facilities in your region?

    - My VAMC has experienced a CRE/CP-CRE outbreak in the last year
    - My VAMC is located in a region known to have a high prevalence of CRE/CP-CRE
    - CRE/CP-CRE is increasing at my facility
    - We have not seen any CRE/CP-CRE cases at our VAMC
    - Incidence of new CRE/CP-CRE patients is ~< 1 per month at my VAMC
    - Incidence of new CRE/CP-CRE patients is ~ ≥ 1 per month at my VAMC
    - None of these apply to my facility

7. Guidelines: Tell me about the guidelines or recommendations your facility follows related to CRE/CP-CRE. Prompts: Which guideline (VHA, state, CDC)?

    a. How would you describe your role in implementing the guidelines?
    b. How did you initially learn about the guidelines? Who is responsible for dissemination at your facility?
    c. What type of training or education did you receive about the guidelines themselves or implementing them?
    d. What was your perception of the education/training you received?
    e. What about others, did they get this training?

f. Who provided it?

g. How is training on the guidelines handled for new staff at your facility?

h. What efforts have been made to engage staff in implementing the CRE guidelines?

i. Based on your experience, do you have any additional suggestions about how to better engage staff in implementing the CRE guidelines?

j. Overall can you tell us whether you would rate guideline implementation at your VAMC as fully/mostly/partially /not at all implemented at your facility. Tell me a little about why you've rated it this way.

k. How would you describe the impact of the CRE guidelines on your facility's infection control practices?

l. Do you have a local policy that's approved (or in development)? If yes, tell us about the process. If no, do you plan on one? Can you tell us anything about what you think might be holding things up?

Screening Questions

8. Routine surveillance: Does your facility routinely perform active surveillance for CRE/CP-CRE? (Prompt: Urine/blood samples vs. rectal swabs?)
   a. If your VAMC does routine surveillance, what is the high risk screening criteria at your facility? (Prompt: those admitted to long term care beds, SCI, ICU, those admitted from other high risk facilities?)

   b. If your VAMC does perform routine surveillance, where do you think that the majority of your CRE/CP-CRE cases come from?
   (Prompt: Inside/outside your VAMC or catchment area? If inside, Long Term Care, SCI, etc.)

8a) What anatomic locations do you typically swab for CRE/CP-CRE surveillance?
   a. What information is provided to patients or families about why they are being screened?
   b. Any issues with obtaining patient consent? Cohorting?

8b) If your facility does not currently screen high risk patients, can you tell me what you know about your VAMC's plans?

Processes related to admission of a CRE+ patient

9. What is your role as the MDRO coordinator when a patient tests positive for CRE/CP-CRE? Please tell me what happens when a patient is identified as CRE/CP-CRE positive.

   a. Are other patients in the surrounding area/unit screened as a result?

   b. Are CRE/CP-CRE positive patients placed in a private room or are they cohorted with other patients?

Please tell me about this.

c. How is information about the positive culture typically shared with other clinical staff?

d. Is there an alert/flag in the patient's medical record? If yes, how long does the alert stay there?

10. Please tell me about the process for admitting a patient from another facility who has been identified as CRE/CP-CRE positive.
    a. How does the transferring facility communicate that information to you?
    b. How do VA facilities and non-VA facilities compare in terms of providing information on CRE/CP-CRE positive patients they transfer to your facility?
    c. Any Best practices you can share with us?
    d. When you discharge a patient to *another facility,* what steps do you take to let that facility know about the patient's CRE/CP-CRE status?
    e. Any best practices you can share?
    f. Any difference in the process if the patient is being transferred to a non-VA facility?

11. At your facility, what type of information do patients receive about CRE and MDROs?
    a. How are patients educated about CRE?
    b. What aspects of the patient education are good and what aspects could be improved?

Attitudes & Leadership

12. How would you describe attitudes at your facility around the prevention and management of HAIs (in general)?

    a) How would you describe staff attitudes regarding CRE prevention? (E.g., Is the CRE guideline something they see as an issue relevant to your facility? Is it something they see as important?

    b) What is the role of leadership in identification, surveillance and control of CP-CRE at your facility?

    c) In what ways has VA leadership shown support/provided support for implementation of the CRE guidelines? What about local leadership at your facility or within your unit?

    d) Who are the key individuals at your facility engaged in implementation of the guidelines? (e.g., Infection Control, Quality)?

13. Who else at your facility should we talk with possibly stewardship, infection control, additional lab, or medical staff for further interviews?

Stewardship

14. Do you know whether your facility have any restrictions in place for carbapenem use? If so, what are they?

15. In what way does your facility's Antimicrobial Stewardship Program work with laboratory staff and Infection Control in implementing the CRE/CP-CRE guidelines?

Lab INTERVIEW QUESTIONS ON CRE GUIDELINES
VA QUERI CARRIAGE Program
Interview about Laboratory and Infection Control Practices Related to Carbapenem-resistant Enterobacteriaceae (CRE)

These interviews are focused on understanding infection control practices related to CRE in your facility. The VHA 2017 Guideline for Control of Carbapenemase Producing-Carbapenem Resistant *Enterobacteriaceae* (CP-CRE) is focused on CP-CRE, but we are also interested in learning about practices related to the identification, surveillance, and control of all CRE. The interviews will help us to understand current implementation of the VHA CP-CRE guidelines and to identify best practices and recommendations to support implementation across VA facilities. You are being asked to participate because of your role in CRE surveillance practices and procedures.

Demographics

16. What is the title of your position?

17. How long have you been working in the VA?

18. How long have you been working in this position?

19. Gender:

Guideline Related Questions

20. How did you 1st become aware of the guidelines or recommendations your facility follows? Did you attend any in-person VA-sponsored training on CRE or did you review online materials? If you viewed them online, did you have any questions about the guidelines? Did anyone go over these policies with you after you reviewed?
    a. Who is the person at your facility who is responsible for distributing information about the guideline? In your experience, how is that information distributed? What is your role in implementation of the CRE guidelines?
    b. What is your perception of the information you received? What do you think could be improved about how the information is distributed? What do you think is the best format for delivering this information? Are the right people getting the necessary information?
    c. What tasks do you think are necessary to complete what is required of the guidelines? Are the tasks that are required clear? How would you describe your knowledge of and experience with the CRE guidelines as far as whether you can successfully complete the tasks required in the guidelines? Can you explain your response?
    d. How confident are you that you can implement your part of the guidelines?
        i. If not fully confident, what barriers/facilitators have you encountered that have left you feeling less confident about adhering to the guidelines related to CRE/CP-CRE?

21. Please tell me a little bit about your facility's experience with CRE/CP-CRE. To what extent is CRE a problem at your facility? Other facilities in your region? What about other facilities in your community?
    a. My VAMC has experienced a CRE/CP-CRE outbreak in the last year
    b. My VAMC is located in a region known to have a high prevalence of CRE/CP-CRE
    c. CRE/CP-CRE is increasing at my facility
    d. We have not seen any CRE/CP-CRE cases at our VAMC
    e. Incidence of new CRE/CP-CRE patients is ~$<$ 1 per month at my VAMC
    f. Incidence of new CRE/CP-CRE patients is ~ $\geq$ 1 per month at my VAMC
    g. None of these apply to my facility

22. Have you had any issues with CLSI standards updates and changes to your automatic testing system? What mechanisms/work arounds are in place?

23. Do you do microbiology testing for any other VAMCs? Which sites or facilities? Does this include CRE?

    Do you send your samples to any other VAMC or reference lab for microbiologic testing? Which site or facility? What about for CRE?

Testing on CRE/CP-CRE

Please tell us about your process for testing for CRE?

When you find CRE, are you confirming if it's a carbapenemase producer?

Which method(s) does your laboratory use to confirm that a CRE isolate produces a carbapenemase? (Modified Hodge Test (MHT), CarbaNP, PCR-based detection from the initial specimen or culture such as KPC, NDM, IMP, VIM, OXA-48)

24. Where do you document the result of it being a carbapenemase producer? Prompts (is it in the comments section of Microbiology? Is there a specific place in the laboratory records that it is placed in?

    If Not confirming that it's a carbapenemase: What are the barriers that are preventing you from performing confirmatory testing? (testing tools, resources). What would facilitate your ability to perform these tests?

25. Have you encountered or do you think you might encounter any environmental barriers/facilitators when trying to accomplish the tasks required? (i.e. lab equipment issues, testing tools and resources, development of local work-arounds?)

26. Does your facility have any "best practices", if any, that can be implemented to improve the laboratorian processes for CRE/CP-CRE?

27. What level of priority would you say that implementation of the CRE guidelines is at your facility (high vs. low)? Tell me why you say that.

28. Overall can you tell us whether you would rate guideline implementation at your VAMC as fully/mostly/partially/not at all implemented at your facility. Tell me a little about why you've rated it this way.

Teamwork/Communication

29. How does the lab communicate information about a patient testing positive for CRE/CP-CRE? (To whom is that information communicated: attending physician, infectious disease physician, CDC, unit nursing manager? Or they have a protocol about communicating these results? Is the information shared beyond those the lab contacts? Who communicates it and how is that information further communicated?) What issues, if any, are there with this process that could be improved? Best practices? [Inner setting: Structural characteristics]

30. What does teamwork look like in handling CP-CRE related infections?
    a. Can you describe instances where you have experienced good teamwork around the management and control of CP-CRE related infections? Any more examples? Are there examples where there were challenges to teamwork?

Attitudes & Leadership

31. How would you describe attitudes at your facility around the prevention and management of HAIs (in general)? How would you describe staff attitudes regarding CRE prevention? (E.g., Is it something they see as an issue relevant to your facility? Is it something they see as important?)

32. What is leadership's role in identification, surveillance and control of CP-CRE at your facility?

33. In what ways has VA leadership shown support/provided support for implementation of the CRE guidelines? What about local leadership at your facility or within your unit?

34. Who else at your facility should we talk with possibly stewardship, infection control, additional lab, or medical staff for further interviews?

Stewardship Specific Questions

1. Does your facility have any restrictions in place for carbapenem use? If so, what are they?

2. In what way does your facility's Antimicrobial Stewardship Program work with laboratory staff and Infection Control in implementing the CRE/CP-CRE guidelines?
