# Supplementary material for: Evaluation of carbapenem-resistant Enterobacteriaceae (CRE) guideline implementation in the Veterans Affairs Medical Centers using the consolidated framework for implementation research
Source: Implement Sci Commun. 2021 Jun 29;2:69. doi: 10.1186/s43058-021-00170-5 (PMC8243642; doi:10.1186/s43058-021-00170-5)
Supplement: Supplementary file 3 — Additional file 3. CFIR Constructs and Construct Definitions. [file 43058_2021_170_MOESM3_ESM.docx]

| **CFIR Constructs** | **Construct Definitions** |
| --- | --- |
| **Domain: Intervention Characteristics** | |
| Intervention Source | Using VA Guidelines [y/n] |
| Evidence Strength and Quality | High confidence in CRE guidelines (or guideline development process) |
| Relative Advantage | Strategies used until (CLSI, VA/CDC) guidelines came out (workarounds) |
| Adaptability | Development of local policies & templates |
| Trialability | Discussion of implication for large scale testing of specimens and whether guideline would ever be modified |
| Complexity | Perceived difficulty of implementation, reflected by duration, scope, radicalness, disruptiveness, centrality, and intricacy and number of steps required to implement |
| Design Quality and Packaging | Dissemination of guidelines and compatibility between VA, CDC, and/or state guidelines |
| Cost | Cost of specimen collection (including new equipment or testing supplies), Lab/staff time |
| **Domain: Outer Setting** | |
| Patient Needs and Resources | Description of strategies/materials used to educate/engage Veterans and their families |
| Cosmopolitanism | Strategies for working out conflicts or differences in guideline requirements |
| Peer Pressure | When a VAMC has already developed/implemented its own policies this creates pressure for other VAMCs to do this |
| External Policy and Incentives | External reporting (e.g., State, NHSN, XDRO Registry, IPEC, VISN) |
| **Domain: Inner Setting** | |
| Structural Characteristics | Bed/room availability for isolating CRE+ patients (e.g., Specialty/High Risk Units, Multibed rooms, Isolation rooms) |
| Networks & Communications | Local infrastructure for national guideline or training material dissemination (e.g., CRE-related conference calls to train staff) |
| Culture | Infection Control policies already implemented locally in anticipation of new MDROs |
| Implementation Climate | Shared beliefs about effectiveness of CRE guidelines in improving health for Veterans |
| Tension for Change | The degree to which stakeholders perceive the current situation as intolerable or needing change. |
| Compatibility | Implementing CRE guidelines fits their view of VA as a responsive citizen and national leader in IC, CRE is just one more HAI (RNs know they will have to address even if they haven't seen it already) |
| Relative Priority | CRE will be treated as seriously as other HAIs |
| Organizational Incentives/Rewards | While actual sanctions seem far off, failure to implement CRE guidelines diminishes VAMC status in their own eyes |
| Goals and Feedback | Strong expectation of all staff participating in clear and open communication around CRE |
| Learning Climate | Strong expectation of participation in all guideline related activities; can openly say something isn't working or discuss shortcomings (safe to see something, say something) |
| Readiness for Implementation | Clear expectations that guidelines must be implemented (even if they haven't done it yet) |
| Leadership Engagement | Commitment, involvement and accountability of local leaders and managers with the guideline implementation |
| Available Resources | Money, equipment, testing supplies, training, education, isolation space, staff time, IT support, and previous work-arounds to facilitate guideline implementation are available |
| Access to Knowledge & Information | Guideline or training materials (policies, templates, cheat sheets) locally disseminated to relevant stakeholders at each facility |

| **Domain: Characteristics of Individuals** | |
| --- | --- |
| Knowledge & Beliefs about the Intervention | Positive or negative attitudes about implementing CRE guidelines |
| Self-Efficacy | Confidence in understanding and implementing CRE guidelines |
| Individual Stage of Change | Expectations or metrics about CRE implementation |
| Individual Identification with Organization | Their self-evaluation of their relationship with and commitment to their unit, VAMC or VA generally |
| Other Personal Attributes | Their self-evaluation of their relationship with and commitment to their unit, VAMC or VA generally OR Congruence between their ability to implement CRE guidelines and their own professional expectations |
| **Domain: Process** | |
| Planning | VAMC has an existing structure/process for Infection Control and lab testing for other MDROs |
| Engaging | High confidence in their or their team's ability to implement at their VAMC [y/n] |
| Opinion Leaders | Experience/confidence in existing strategies to implement the guideline (I would call chief of staff if there were $$ concerns) |
| Formally Appointed Internal Implementation Leaders | Existing structure (units/departments/committees/resources) for IC/lab testing for other MDROs |
| Champions | “Individuals who dedicate themselves to supporting, marketing, and ‘driving through’ an [implementation],” overcoming indifference or resistance that the intervention may provoke in an organization |
| Executing | High enthusiasm/self-efficacy for implementing CRE guidelines/policies |
| Reflecting and Evaluating | Shared understanding of their VAMC's progress in implementing the CRE guidelines within VISN and dual care (e.g. academic facilities and local hospitals) |
| External Change Agent | Individuals who are affiliated with an outside entity who formally influence or facilitate intervention decisions in a desirable direction. |
| **Open Codes** | |
| **Open Codes** | **Code Definition** |
| Barrier | Any barrier discussed |
| Facilitator | Any facilitator discussed |
| Feedback &Recommendations | Any feedback/recommendations discussed. |
| Best Practices | Refers to something that is an innovative approach to implementation or guideline recommendations. |
| Patient Characteristics | Screening for CRE (e.g., LTC, SCI, Acute Care/ICU, Community/University, Transplant Unit) |
| Communication Breakdown | Discussions of team communication or breakdowns |
| CRE Reported Incidence | Positive cases or if they haven't seen any cases |
| Infection Control Team | How the Infection Control team involvement in guideline process |
| CRE Guideline Implementation | Guidelines are implemented at their VAMC |
| Nursing | Nursing staff receipt of CRE information |
| Provider | Physicians, NPs, PAs receipt of CRE information |
| Staffing Changes | Comments regarding "staffing issues" |
| Unions | Discussions of unions (e.g., approvals) |
| Cohorting | Confidence and self-efficacy cohorting patients |
| Antimicrobial Stewardship | Antimicrobial Stewardship team involvement in guideline process |
