# Supplementary material for: Evaluation of carbapenem-resistant Enterobacteriaceae (CRE) guideline implementation in the Veterans Affairs Medical Centers using the consolidated framework for implementation research
Source: Implement Sci Commun. 2021 Jun 29;2:69. doi: 10.1186/s43058-021-00170-5 (PMC8243642; doi:10.1186/s43058-021-00170-5)
Supplement: Supplementary file 4 — Additional file 4. MPC Interview Case Memos: Support and Enhancement of VA Guidelines to Prevent the Spread of Carbapenemresistant Enterobacteriaceae (CRE) Qualitative Interview results. [file 43058_2021_170_MOESM4_ESM.pdf]

Support and Enhancement of VA Guidelines to Prevent the Spread of Carbapenem-resistant Enterobacteriaceae (CRE) Qualitative Interview results

Veterans Health Administration
Combating Antimicrobial Resistance through Rapid Implementation of Available Guidelines and Evidence (CARRIAGE) QUERI Program

**CARRIAGE QUERI CRE Study Team**
Charlesnika T. Evans, PhD, MPH
Brian Bartle, MPH
Margaret Fitzpatrick, MD, MS
Cassie Goedken, MPH
Marylou Guihan, PhD
Linda Poggensee, MS
Swetha Ramanathan, MPH
Heather Reisinger, PhD
Katie Suda, PharmD, MS
Amanda Vivo, MPH

The goal of the CARRIAGE QUERI program is to address the growing public health crisis of anti-microbial resistance through strategies that support the uptake of new and existing evidence-based practices, policies, and programs targeting the improved antibiotic use and prevention of healthcare-associated infections.

QI Project Summary

To address the growing threat of Carbapenem Resistant *Enterobacteriaceae* (CRE), VA's Multi-Drug Resistant Organism (MDRO) Program Office disseminated guidelines in 2015 and updated them in 2017, with a focus on Carbapenemase Producing-Carbapenem Resistant *Enterobacteriaceae* (CP-CRE). The guidelines aim to increase surveillance and standardize CRE screening and reporting, provide guidance on testing procedures for clinical cultures, and optimize infection prevention in acute areas and VA nursing homes. Qualitative interviews were conducted with staff to identify perceptions about implementation of the VHA 2015/2017 guidelines. The goal of this project was to better understand the contextual factors, challenges, and best practices influencing implementation of the CRE/CP-CRE guidelines within VAMCs.

Methods

Sample selection

- Used CRE incidence quartiles (derived using CDW data with 2015 definitions) to select large and small VAMCs (n=29).
- Oversampled facilities indicating that their VAMC was currently screening for CRE.

Participants

- 43 VA staff were interviewed, including microbiology laboratory staff (47%), MPCs (35%), infection control nurses (12%) and physicians (6%).

Data analysis

- Interview quotes (n=841) were coded as positive/negative and categorized into themes and/or best practices (verified by clinical study team members).
- Fisher's exact test was used to compare themes for screening/non-screening and with CRE/no CRE sites.

Results

- Main interview themes included: 1) leadership engagement, 2) team communication, 3) relative priority, 4) available resources (e.g., IT support, staffing), 5) access to knowledge & information.

This report describes interview results and highlights potentially modifiable factors that may aid successful VA CRE guideline implementation.

1. **National & Local Leadership Engagement:** Leadership involvement, commitment, and accountability with the guideline implementation**.**

(+) [If we tell leadership what's needed], they'll say okay and get it for us. If we started seeing more CREs, and we need [new testing equipment], they'll find the money to support it.

(+) Laboratory [leadership handles all] equipment needs … and any new assays. [IC]

(-) [Leadership] allowed us to do a CPRS flag and … to send out confirmation testing and … set up send out protocols. However, it takes a long time to get this much done. [MPC]

Best practices

**[BP][Our] Medical Director is also very involved in Infection Control and Infectious Disease… [and] works with [us] on projects that arise. [MPC]*

Discussion:

- Participants described different levels of leadership involvement (VISN, facility, Infectious Disease, Laboratory, Infection Control team, Antibiotic Stewardship Programs [ASP]), down to the roles of individuals (e.g., Infectious Disease [ID] physicians) and the role they can play in accessing resources or addressing concerns.
- Even at sites where administrative leaders were described as less involved in the day-to-day infection control [IC] activities, every participant described the need for leadership involvement (sometimes at critical time periods or to address key aspects of the implementation).
- Even strong leaders might struggle with entrenched bureaucracies (e.g., to secure funding to purchase expensive lab testing equipment, hiring) as shown in the comments above. Leadership involvement at one or more levels (clinical or administrative) was described as necessary to succeed in guideline implementation.

VAMCs with active CRE screening (vs. not screening) reported significantly more leadership involvement in implementing CRE policies, 100% vs. 68.2%, (p=0.002).

2. **Communication:** Discussions of team communication (+) or breakdowns (-).

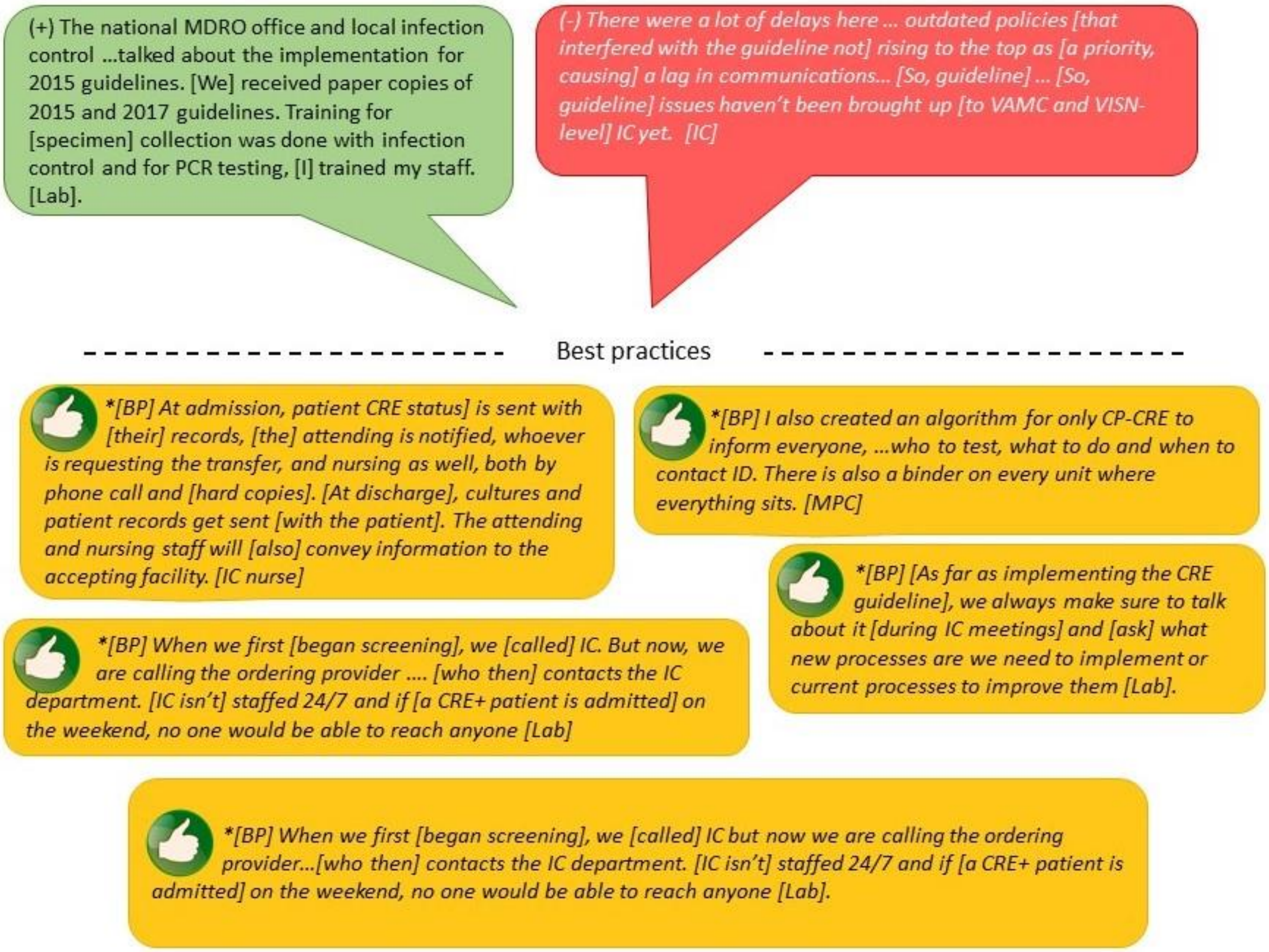

Discussion:

- Sites reporting at least 1 CRE+ patient also described using multiple strategies for disseminating and implementing the guideline (e.g.,
  - creating an algorithm for screening/communicating CRE+ findings,
  - screening protocol binders located in each hospital unit,
  - printouts of overnight test results,
  - email groups, phone trees or voicemails and
  - online pamphlets staff can easily access/print out if Infection Control staff isn't available, etc.).

VAMCs with No CRE report more team communication breakdowns than sites with CRE: 100% vs. 80.8% ($p=0.02$).

3. Relative Priority: CRE is treated as seriously as other health associated infections (HAIs).

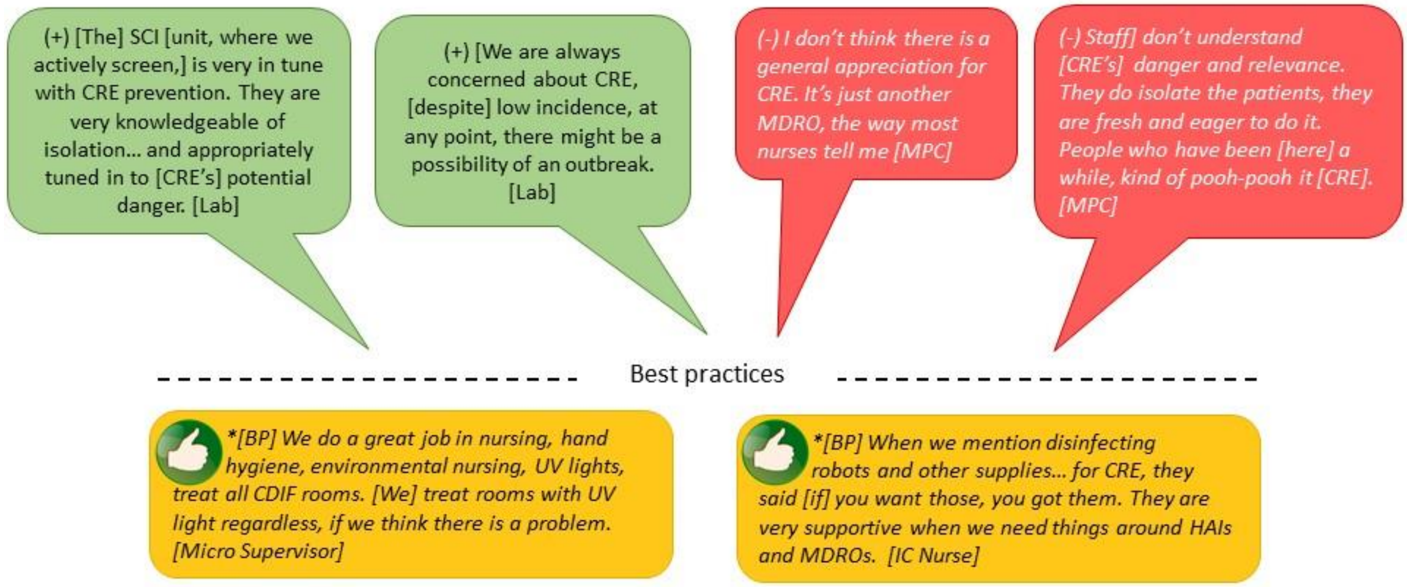

Discussion:

- Although VA administrative data indicates that about 70% of VAMCs had at least 1 CRE+ specimen and/or are in a region with high CRE rates, most VAMCs report not currently screening due to low CRE incidence.
- Only one site reported actively screening patients in all high risk CRE categories. Other screening sites focused on admissions/transfers from the following areas: SCI (N=19), LTACs (N=19), community /university (N=15), acute care/ICU (N=8), and/or transplant (N=2).
- In 2017, CDC indicated that CRE/CP-CRE has been reported in every state, therefore suggesting even VAMCs reporting no CRE+ cases may experience cases in the future. It is critical that these facilities are prepared.

Sites screening for CRE more likely to report that CRE is treated as seriously as other HAIs (when compared to sites not screening for CRE) 91.7% vs. 55.6% ($p=0.01$).

4. Access to Knowledge and Information: CRE Guideline is disseminated to relevant stakeholders at each facility.

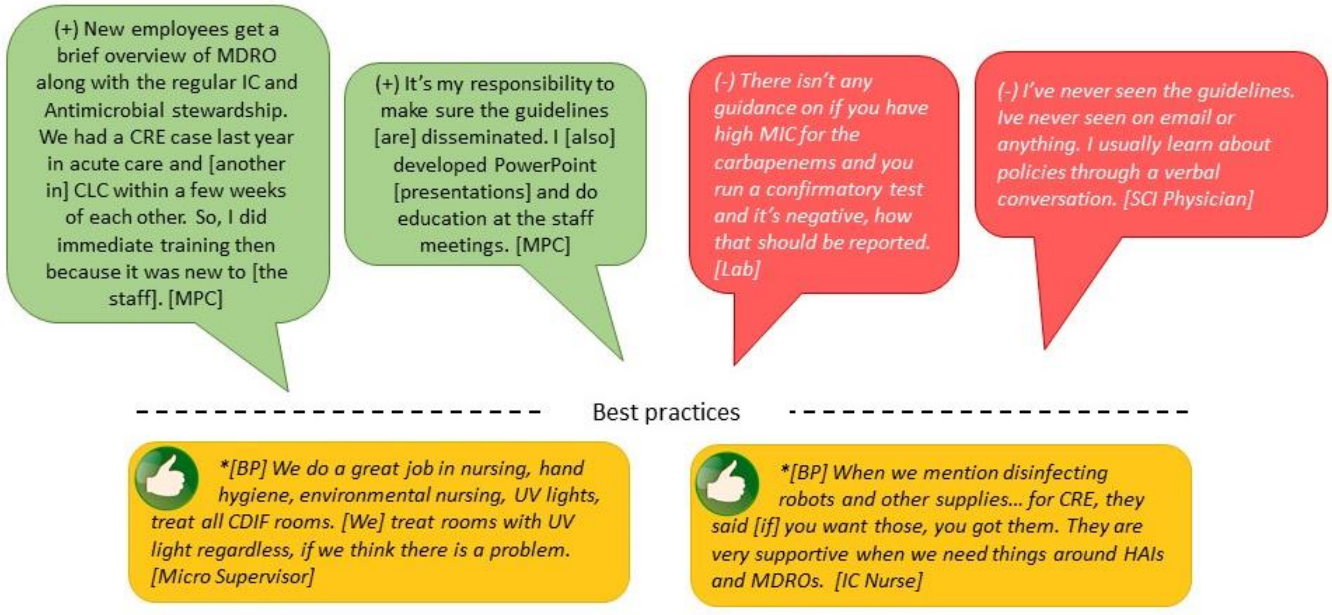

Discussion:

- Implementing CRE guidelines requires access to:
  - timely PCR testing and
  - staff education/training.
- Sites experiencing CRE describe obtaining timely access to PCR testing, conducting staff education and training.
- They also report strategies to develop or adapt existing policies/procedures/protocols to address:
  - active screening of high-risk patients (including SCI patients who are not addressed in the guideline) at admission
  - communicating patient CRE status to all relevant parties (e.g., including the ordering provider),
  - site specific plan for potential long-term isolation,
  - enhanced staff and patient educational material including obtaining consent for CRE specimen collection,
  - enhanced tools (e.g., CPRS screening, admission/discharge templates and
  - enhanced reporting to facilitate clinical decision making).

Sites with CRE report better access to knowledge and information (compared with sites reporting no CRE) 88.9 % vs. 36.8% (p=0.02).

5. Available Resources: Funding, lab equipment/testing supplies, training/educational materials, isolation space, staff time and IT support to facilitate guideline implementation are available.

(+) IT support and staffing: We have CPRS [discharge] template [to communicate CRE status] and nurses [also] call the accepting facility and give a [verbal] report. [MPC]

(+) Timely lab reporting: The [CRE testing] cartridges last about 4-6 months,(...) and if I only get 3 [CRE cases] within those 6 months, then I have to throw the other 7 cartridges away. [By comparison, the University lab can provide] results in] ... about 24 hours.[Lab]

(-) Lab testing equipment & training: [New lab equipment has to be purchased and time has to be allocated for staff to be trained and be proficient.] it's hard to add new testing in the VA. [Lab]

(-) Staffing: [We] had a lot of [staff] turnover in infection control for a long time, and when you have less experienced [people with] less training, then that's a challenge. [IC]

(-) Educational materials/ Staffing: It would be nice to have [more educational] materials [and staff release time for CRE-related training]. [MPC]

(-) Isolation beds: [Our] spinal cord [unit][has only] four bed room[s] [individual isolation rooms are not available]. That's an infection control issue if someone is [CRE] positive. [MPC]

(-) IT support: The handoff communication is verbal and the transfer note doesn't [routinely address the patient's] isolation status. When I call the new unit, [unless] they check their Theradoc ... they won't know. [MPC]

Best practices

**[In addition to] unit-based training, [we] developed poster boards [for] the Infection Control week. After [the] initial training, physicians were still ignoring and bypassing order sets. [So] posters [were hung] in meeting rooms and any other rooms where physicians did their documentation. They finally are getting numbers that are representative. [ID Chief]*

**One thing that worked well for us is (despite staff turnover and disinterest) is having a[n] infection control champion on each unit, where there is more intensive education with that person. That person then dissemination to their staff or they are the go-to person. [MPC]*

Discussion:

Sites successfully implementing CRE guidelines describe strategies they use to:

- enhance dissemination of CRE educational materials to providers (e.g., posters and train-the-trainer in-services),
- develop PCR testing capability (e.g., by purchasing new equipment and/or identify an outside lab with adequate turnaround time)
- ways to systematically communicate patients' CRE status to all relevant parties (strategies described in #4 above)

Sites not screening for CRE are significantly more likely to report fewer available resources (compared to sites screening for CRE): 81.7% vs. 45.1% ($p<0.0001$).

MDRO Program Office Resources:

VHA 2019 CP-CRE Toolkit:
https://vaww.mrsa.va.gov/Carbapenem_resistant_Enterobacteriaceae_CRE.asp

CDC Antimicrobial Resistance Laboratory Network (ALRN):
2019 toolkit appendix D, p. 19

CPRS Interfacility Transfer Form:
CRE guideline, appendix V, p. 18

CRE educational brochures for staff and patients/families:
https://vaww.mrsa.va.gov/docs/CP-CRE_brochure_2019.pdf

CRE educational materials:
https://vaww.mrsa.va.gov/Education.asp.

CRE risk factors facility screening criteria:

-2019 toolkit, Facility characteristics: 2. p. 19

- 2019 toolkit, 3. Possible high-risk candidates for patient screening

Obtaining patient consent to collect CRE specimens: 2019 toolkit, p. 10-11. 4 b

Collection of CRE specimens: 2019 Toolkit, p. 11, 4c-e
